# Responding to the learning crisis: Structured pedagogy in sub-Saharan Africa

**DOI:** 10.1016/j.ijedudev.2024.103095

**Published:** 2024-09

**Authors:** Benjamin Piper, Margaret M. Dubeck

**Affiliations:** a Bill and Melinda Gates Foundation, Kenya; b RTI International, United States

**Keywords:** Structured pedagogy, Foundational literacy and numeracy, Teacher

## Abstract

• Describe the history of structured pedagogy and summarize research on its use to improve foundational learning.
• Define structured pedagogy as a coherent package of instructional materials, intial training, and ongoing support.
• Present a solution to address low learning levels in formal education settings.

## 1 Introduction

**Fig. 1:**
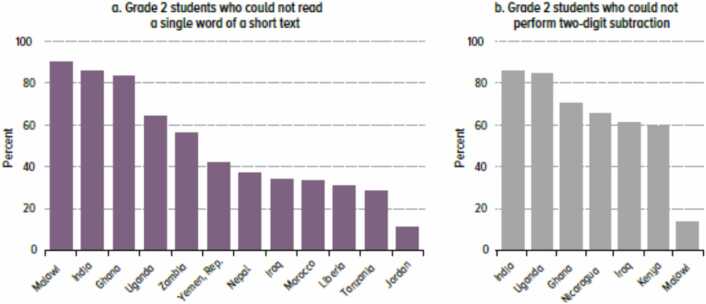
Percentage of Grade 2 students in low- and middle-income countries unable to perform basic literacy or numeracy tasks (World Bank, 2018).

Learning outcomes are disastrously low for most children in many low- and middle-income countries (LMICs). Children are simply not learning basic literacy and numeracy skills and are therefore less likely to be substantial economic contributors in a modernized economy. Lower primary grades are characterized by wastage and churn due to poor management and ineffective instruction (Crouch et al., 2020). In some LMICs, half or more of the grade 2 population is unable to read a single word of a short text or do two-digit subtraction (World Bank, 2018), as shown in Fig. 1. This limits the development of human capital needed for LMIC economic growth. Pritchett and Viarengo (2021) show that only a handful of children in the entire country of Zambia can read at a global proficient level (Program for International Student Assessment (PISA) Level 4 or above).

The World Bank’s (2019) data suggests that approximately 53 % of LMIC children are suffering from learning poverty, and the results are even higher in sub-Saharan Africa, at more than 80 % for both illiteracy and innumeracy. Given these disastrous results, urgent action on educational improvement interventions and reforms are required. Unfortunately, low learning levels are not easy to improve. Poor instructional methods are used in a preponderance of lower primary classrooms. This is partially due to a mismatch of the taught curriculum and the skills of children (Muralidharan and Singh, 2019), the substantial utilization of colonial languages like English (Piper et al., 2016), and the expectation that it is the curriculum content that should be taught rather than children (Pritchett, 2013). Some studies suggest that children are disserved by underutilization of instructional time due to absence and tardiness (teacher and student) (Shuh-Moore et al., 2011). The typical teacher-centered instruction that does not model or provide opportunities for each child to engage with the content is ineffective at teaching literacy and numeracy skills (Commeyras and Inyega, 2007). Teachers in many contexts remain hesitant to the instructional fads that come and go in education (Cuban, 1987), and small-scale experiments are difficult to scale up to affect a large number of children (Muralidharan and Niehaus, 2017) and they are not always successful when they are scaled (Muralidharan and Singh, 2020). Evidence that just adding instructional is not the solution comes from an analysis of reading outcomes in 87 countries across 50 years which found that education quality has stagnated or declined in both South Asia and sub-Saharan Africa (Le Nestour et al., 2022). It is in this context that many LMIC governments, experimenting with various learning-improvement solutions, have attempted to implement structured pedagogical interventions to improve the quality of foundational literacy and numeracy instruction.

We define structured pedagogy as a specifically designed, coherent package of investments that work together to improve classroom teaching (Piper et al., 2021). In LMICs, the typical structured pedagogy program includes (1) student books and materials, often at a 1:1 ratio, (2) teachers’ guides that provide daily lesson plans for teachers with some level of structure and specificity, (3) teacher training designed to introduce and reinforce specific skills in teaching the lessons, and (4) continuous support to teachers implementing the structured pedagogy program, often including instructional coaching and teacher communities of practice. These four components, carefully integrated, are well-supported in evidence on how children learn foundational skills. The Global Education Evidence Advisory Panel (2023) argues that structured pedagogy literacy programs include careful phonics-based scope and sequences.

Aiming for improved education outcomes via structured pedagogy should be the priority and not considered an extra. This paper investigates structured pedagogy’s ability to be a solution to the learning crisis in the following way: Using resources developed to support policymakers and donors to develop effective structured pedagogy programs we investigate the history of structured pedagogy and describe the characteristics and structures of structured pedagogy programs currently being implemented. Next, we present data on the impacts that structured pedagogy programs have had on learning outcomes in rigorous designs. After the results, we present recent findings regarding ways in which effective structured pedagogy programs have been implemented, drawn from Piper et al. (2021) and Stern et al. (2021). We close with a discussion of the limitations of structured pedagogy programs and conclude with suggestions for the sector and requests for new areas of research.

## 2 History of structured pedagogy

Structured pedagogy has been in existence in various forms for centuries. Its uses across time share several defining features, such as a system for applying some control to the instructional content, becoming progressively more difficult, and providing teachers with guidance on how to support student learning. Variation in the design of specific structured pedagogy programs over time is seen in the amount of support and instructional choices granted to teachers. This section presents a condensed history of the utilization of structured pedagogy programs across the world.

The Ethiopian Orthodox Church used structured approaches to teaching learners the Ge’ez scriptures for hundreds of years (Wagaw, 1979). In the 1600s, curriculum materials directed European schoolmasters in what and how to teach (Bauml, 2015). In 1830s Germany, the Froebelian approach was highly structured, with explicit instructions and training details (Beatty, 2011). At a similar historical moment in the United States, the increase in public-school access created a need for more standardization (Bauml, 2015; Glatthorn, 2018) which was partially addressed by McGuffey Readers, which were widely used from 1836 to 1920 (Neem, 2018, Bohning, 1986). These materials included features that find echoes in structured pedagogy programs today, such as the repetition of content areas that gets progressively more difficult and specific guides for teachers. In the early 1900s, Montessori schools began in Italy using highly specified set of activities, materials, and methods (Beatty, 2011).

In the past century the level of support to teachers and prescription in materials reflected the education priorities of the time. In the 1920s and 1930s instructional materials provided activities, motivation tips, and discussion ideas and by the 1940s it was more common to find images of student work embedded in teacher guides (Woodward, 1986). In the mid-20th century, Piaget's work on developmental levels was influential and still serves as the theoretical foundation for building on existing knowledge to teach new content in many contexts.

In the mid-20th century, the Tyler rationale (Fogarty, 1976) described four concepts that persist in instructional materials to support teachers: (1) purpose or objectives, (2) suggested experiences to achieve the objectives, (3) organization for efficiency, and (4) guidance on evaluating learning experiences (i.e., informal assessment). Materials emerged organized around these concepts to accommodate teacher abilities. Many post-colonial education systems in sub-Saharan Africa used various types of basal readers to drive literacy skill development, and pedagogical methods focused on adherence to utilizing these materials daily.

In the 1960s, partially in response to a concern for global competition and responding to poverty to elevate outcomes for more students, instructional materials in the United States shifted from broad to discrete skills (Woodward, 1986). For example, DISTAR (a predecessor to Mathematics Mastery and Reading Mastery) used explicit directions and lengthy scripts (Beatty, 2011), and contemporary publishers created similar materials (Fitzgerald et al., 2016).

In the 1980s, to further influence school reform, scope and sequences expanded to include hundreds of discrete skills (Woodward, 1986). One program, Success for All, was highly structured with monitoring and various mechanisms to help teachers use the scripted program effectively (Beatty, 2011). In the early 2000s, structured pedagogy use in the United States was strengthened following the passage of the No Child Left Behind Act (2001), which required states receiving Reading First funding to have a program that was scientifically based and included the essential components of reading outlined by the US National Reading Panel (NICHD, 2000). This requirement was interpreted as a packaged reading program, and 97 % of the funding went to instructional materials and training (Lenski et al., 2016).

At the same time, other national calls for increased standardization and structure were seen in the United Kingdom with the influential Rose Report (Rose, 2006) and in Australia (Rowe, 2005). At the turn of the 21st century, under the National Literacy Strategy, the United Kingdom mandated structured pedagogy, and its influence was realized in just four years, when the percentage of students across the country achieving target literacy levels rose 12 percentage points from 62 % to 74 % (Mourshed et al., 2010). By 2010, nearly two-thirds of all U.S. elementary schools were using a core reading and math program. In the decade since, external comparisons and transparency of curriculum packages are increasingly available (IES, 2020; Nebraska Department of Education, 2020).

In other regions, Singapore in the 1980s mandated a more structured approach leading to coordination of the national curriculum, syllabus, assessment, teaching practices, teacher guides and textbooks (Marshall Cavendish Education, 2013). Learning outcomes in China and Vietnam have progressed using structured approaches (World Bank, 2018b) while the Shanghai Model balances providing teachers structure and autonomy (Liang et al., 2016).

Recently, international scholars have described the use of a structured approach in LMICs (Kazmi, 2019). The model recommended by the Global Reading Network (Kim and Davidson, 2019) has recommended it for use in programs funded by the United States Agency for International Development. The Learning at Scale and Numeracy at Scale studies identified several structured pedagogy programs in its review of highly effective large-scale interventions (Stern et al., 2021, Stern et al., 2023). Modeling learning loss due to the COVID-19 pandemic, (Angrist et al., 2021) used recent evidence on structured pedagogy to suggest it as a potential intervention to improve learning outcomes in the face of substantial learning loss, particularly loss focused on the lower end of the economic and learning distribution.

Throughout the history of its use, structured pedagogy has had mixed reactions. Critiques of structured pedagogy in the past have come from both researchers and theorists. For example, those who adhered to Froebel’s method were described as “cult-like”; Montessori practices were “ritualized”; while scripted lesson plans have been labeled reductionist or as contributing to deskilling of teachers (Petrie, 2012, Beatty, 2011, Hodge and Benko, 2014). Other concerns are that teachers and students are being managed and manipulated with too much teacher talk (Brenner and Hiebert, 2010; Woodward, 1986) and insufficient autonomy for teachers to make judgments. Some leaders in low- and middle- income countries argue that structured pedagogical programs are neocolonial, and that teachers and students should have the opportunity to develop their own instructional pathways, including using teaching to create societal change (Freire, 1974). On the contrary, it is clear that there is room for student autonomy within the structured pedagogy umbrella.

Meanwhile, the users of these materials, the teachers, often have a more nuanced reaction to the provided materials. Beginning teachers and those new to the subject of mathematics or reading say the materials give them confidence in the content and the appropriate sequencing (Bauml, 2015; Crawford, 2004). Most importantly, teachers say they like the approach because they see their students learning. The provided content and the suggested activities save teachers preparation time, freeing them to make adjustments and to be more creative (Piasta et al., 2009; Silfverberg et al., 2015). The most typical teacher complaints are that the materials have too much content and do not align with the abilities of all their students (Dewitz and Jones, 2013, Reisboard and Jay, 2013). It appears that structured pedagogical programs have had both supporters and resistors, so any analysis of potential effects should consider adult users and child academic outcomes.

## 3 Characteristics of structured pedagogy programs

As mentioned earlier, we define structured pedagogy as a specifically designed, coherent package of investments that work together to improve classroom teaching. More practically this includes a coordinated, combined approach to teaching that includes student materials, teacher guides with lesson plans, teacher training, and ongoing support. The common elements that are seen in the lesson plans include: (1) direct explanation, (2) modeling (i.e., demonstration), (3) guided practice (i.e., scaffolding), (4) independent practice (i.e., application), (5) formative assessment, (6) discussion, and (7) monitoring (i.e., attending to student response). How those elements are provided by the teacher depends on other characteristics discussed next.

### 3.1 Structured pedagogy’s continuum on the amount of teacher autonomy

**Fig. 2:**
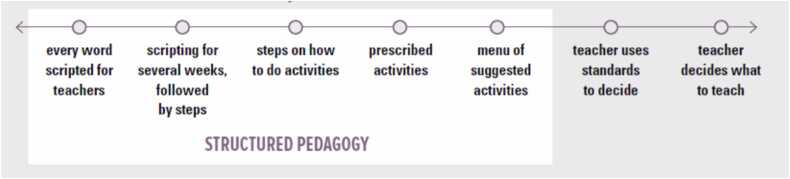
Continuum of teacher autonomy (Piper and Dubeck, 2020).

Structured pedagogy programs vary in the level of prescription and teacher autonomy that they provide in the pedagogical design. Fig. 2 provides a graphical depiction of this spectrum. Structured pedagogy programs are a somewhat broad category and include all the program designs highlighted in white on the left side of Fig. 2. Some programs offer minimal teacher choice and script every word that teachers say. This level of support is helpful when teaching skills that are the foundation to future learning, and the explanation and practice provided is the distinguisher of learning the skill. The script helps teachers to internalize the language so eventually it becomes their own words without skipping explanations or providing too much explanation, both would be ineffective. Yet too much scripting has been shown to have negative impacts (Piper et al., 2018), so it is best reserved for unfamiliar activities. Some teacher guides use just one level of teacher choice (e.g., steps for how to do activities). But other teacher’s guides combine several levels of choice depending on the activity or its frequency. These choices depend on the capacity of teachers, the complexity of the skills to be taught, and whether there is a perception of low achievement and a need for improvement.

### 3.2 Structured pedagogy programs expect teachers to make adaptations

The teachers’ guide is seen as a way to increase consistency in content and to build the skills of teachers. Regardless of the level of choice, some programs expect teachers to maintain strict fidelity to the teacher guide while others expect teachers to make adaptations such as reteaching or differentiation. These are done to meet the needs of the students. However, to expect teachers to make adjustments does not just require permission, it requires more training. Evidence from a multicounty study of teachers' guide use found that the majority of changes made by teachers actually reduced the quality of the lesson (Piper et al., 2018).

Ultimately, a coordinated structured pedagogy program improves teachers' pedagogical knowledge. It strengthens their knowledge of the sequence of skills, relevant practice problems, and understanding students' responses. It frees experienced teachers to add their splash of creativity to the provided lesson (e.g., the Shanghai Model) and it is a logical solution for a teacher new to a subject or a grade level.

### 3.3 Structured pedagogy includes elements that align with research on knowledge acquisition

The science of learning is an interdisciplinary effort that consolidates information from controlled environments (e.g., labs) and field research (e.g., classrooms) to inform educational practice (Meltzoff et al., 2009). The science addresses the way humans acquire knowledge, some of which are realized with structured pedagogy approaches (Deans for Impact, 2015; Horvath et al., 2017), benefiting both students and teachers. Table 1 analyzes correspondence in the research on science of learning and structured pedagogy programs.

**Table 1:** Relationship between the science of learning research and structured pedagogy programs (Piper and Dubeck, 2020).

**Science of Learning**	**Realized in Structured Pedagogy**
Children learn new ideas through connections to what they already know.	A carefully planned scope and sequence (see next section) helps to ensure that students have the prior knowledge they need to master new ideas.
Learning involves moving information from working memory—which has limited capacity—to long-term memory.	Teacher’s guides offer explanations, modeling, and appropriate examples to avoid overwhelming students.
Solving complex problems requires having basic skills available in long-term memory.	Teacher guides include instructional methods (e.g., phonics) that ensure students acquire the basics so they can focus on the more complex skills (e.g., comprehension).
Retention of new ideas requires practice.	Learning materials supply content for both initial acquisition and application of those ideas (reading, writing and orally).
Examples help with learning new ideas, but students can still find it difficult to understand the underlying concept.	Learning materials include both abstract representations (e.g., mathematical calculations) and concrete examples (e.g., word problems) including object manipulation.
Gaining new knowledge and skills requires effective feedback to students.	Teacher training (see next section) and ongoing support help teachers to provide constructive feedback.
Repetition builds automaticity, which frees up thinking for other simultaneous activities.	For teachers, they develop automaticity by using a structured lesson that is predictable which allows them to focus on student learning, their pedagogical choices, and how to improve instruction. For students, with appropriate, applied repetition they develop automaticity for specific skills (e.g. word recognition) which helps in other areas (e.g., reading comprehension).

## 4 Impacts of structured pedagogy programs on learning outcomes

We have shown that structured pedagogy programs are not new, but the growing utilization of these programs in LMICs for foundational learning suggests the need for evidence as to their potential effects on learning outcomes. In this section, we present descriptive analysis of whether these programs have an impact from four areas of evidence.

Structured pedagogy is a relatively large umbrella and includes programs with a variety of designs, in different locations and at a variety of schools. Our first body of evidence is derived from the large number of education meta-analyses published in the 2010s. Banerjee’s et al. (2013) work on post-primary education included evidence of program effectiveness among programs that could be considered structured pedagogy, but several other papers focused on lower primary grades. These include Conn (2014), McEwan (2012), Murnane and Ganimian (2014) and Krishnaratne et al. (2013) which all have studies that show impact on learning. Glewwe et al. (2014) showed that key elements of structured pedagogy were essential to improving learning, and Evans and Popova (2015) show that the definition of successful programs differ but include structured pedagogy programs in their estimates. This set of meta-analyses all include evidence that structured pedagogy programs can work, but the majority of programs were implemented at small scale.

A second area of evidence on the impact of structured pedagogy programs has accumulated since 2015. This more recent evidence was consolidated in Graham and Kelly (2018), Stern and Piper (2019) and, particularly for sub-Saharan Africa, Evans and Mendez Acosta (2020). These three data sources show an increasing number of large-scale structured pedagogy programs that have statistically significant and substantive impacts on learning outcomes. Stern and Piper (2019), compare the magnitude of structured pedagogy programs in LMICs with similar programs in the US. Our analysis suggests that impacts in LMIC are twice as large as the impacts in the US, with an average effect size of Cohen’s .44 SD and .22 SD, respectively. Cohen’s benchmarks would put the .44 SD as a small effect, with the benchmark for moderate effects at .50 SD. Kraft’s (2020) recent work in the US suggests that the effect size breakdowns originally developed by Cohen were inappropriate for the education sector and argues that .20 SD should be considered large. Evans and Yuan (2020) updated Kraft’s (2020) analysis for a sub-Saharan Africa context. They show that the 90th percentile of program impacts on learning in sub-Saharan Africa was .38 SD. Therefore, the .44 SD average impact of structured pedagogy programs in Graham and Kelly (2018) and Stern and Piper (2019) show that, at least for the first wave of large-scale structured pedagogy programs, the average impacts were substantially large, above the 90th percentile on average.

A third area of evidence comes from the Global Education Evidence Advisory Panel convened by the World Bank and the UK’s Foreign, Commonwealth and Development Office evaluated the rigorous evidence to put programs into tiers based on their effect and, critically, their cost-effectiveness (Global Education Evidence Advisory Panel (GEEAP), 2020, Global Education Evidence Advisory Panel (GEEAP), 2023). The panel’s analysis was called the Smart Buys, and it compared programs using learning-adjusted years of schooling (Angrist et al., 2021) rather than effect sizes. The Smart Buys document showed that structured pedagogy programs have substantial impacts on learning, can be effective at large scale, and can do it cost-effectively. Unlike the meta-analyses described above, the Global Education Evidence Advisory Panel Smart Buys document compared structured pedagogy interventions alongside other activities. The newest version of the Smart Buys report placed structured pedagogy as a Great Buy, alongside the Teaching at the Right Level interventions with similar cost-effective impacts on learning at scale (Banerjee et al., 2016).

**Fig. 3:**
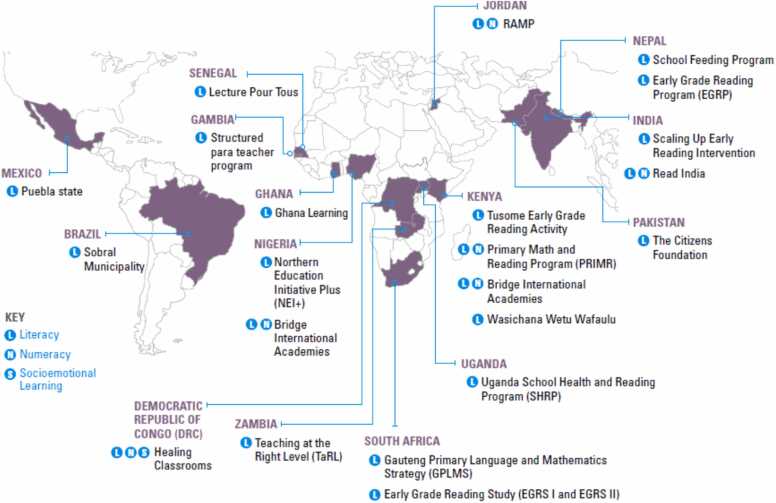
Recent, large-scale, structured pedagogy programs in low- and middle-income countries with rigorous evidence of impact (Piper and Dubeck, 2020).

A fourth area of evidence comes from recent consolidation studies by The Learning at Scale study team, with support from the Center for Global Development. Learning at Scale worked with donors, implementers, and policymakers to identify large-scale interventions with rigorous evidence on improved learning outcomes. The study team identified eight literacy programs as meeting the criteria of both large scale and with large impacts on learning, in this case all foundational literacy. Seven of the eight programs fit into the structured pedagogy umbrella (Dubeck et al., 2019). The Numeracy at Scale team found 3 of the 6 most effective large scale programs in the numeracy area utilized structured pedagogy (Stern et al., 2023). The Science of Teaching research, developed a set of policymaker-facing guides on implementing structured pedagogy. This study identified other large-scale structured pedagogy programs in LMICs with rigorous evidence of program impact. The map in Fig. 3 shows that structured pedagogy programs are improving learning outcomes in several continents, in literacy, numeracy and socioemotional learning, and with a variety of funding sources, including government.

### 4.1 Structured pedagogy programs’ large effect sizes may mask small substantive gains

The four areas of evidence we present above suggests that structured pedagogy programs can have an impact on learning outcomes in LMICs, including at scale. The magnitude of the impacts can be large in terms of effect sizes, but the actual meaningful impacts of these programs can be somewhat misleading. This is due to two factors: how effect sizes are calculated and the large number of children with zeros in their assessment scores. In fact, we show elsewhere that given the low levels of learning in LMICs before the advent of educational interventions such as structured pedagogy, large effect sizes can be associated with modest impacts on learning (Stern and Piper, 2019). Several structured pedagogy programs reduce the proportion of children with very low levels of literacy skills substantially, resulting in high effect sizes, but have relatively small impacts on the overall portion of children who meet government benchmarks for learning outcomes.

## 5 Implementing structured pedagogy programs

We have presented the evidence on the impact of structured pedagogy programs on learning outcomes. This section describes the evidence on how the effective structured pedagogy programs are implemented. First, we discuss how effective structured pedagogy programs work with or alongside government. Next, we present suggestions how the four components of a coordinated structured pedagogy program are designed, beginning with the student and teacher instructional materials followed by the teacher training and ongoing support.

### 5.1 Structured pedagogy programs and government

Large-scale foundational literacy and numeracy programs cannot be effective without government leadership. Learning outcomes are difficult to improve, but when government leads and encourages teachers to adopt the program, some of the initial barriers to implementation can be overcome. This section presents several strategies for working with government that are essential for structured pedagogy programs to be effective, drawn from the Learning at Scale study of eight highly effective large-scale programs (Stern et al., 2021) and from guidance documents for structured pedagogy programs (Piper et al., 2020).

The Learning at Scale study had five findings on why the programs were successful, summarized here. First, the program was a government priority. This meant that the program was more effective because the issue was shared by the intervention program team, whether donor funded or embedded within government, but also that the solution that the program used to respond to the low learning outcomes was agreed to as a government priority.

Second, these highly effective programs were able to set expectations for the intervention and then communicate those expectations throughout the system. Structured pedagogy programs do this differently, but the message that the program is a priority needs to be clearly communicated throughout the system. The national level is insufficient for this, and district or subnational officers played an essential role in sharing these expectations to teachers to attempt to convince them to adopt the structured program elements.

Third, these effective programs monitored implementation of the intervention throughout the system. This moves the importance of the structured pedagogy away from simple communication via circulars and SMS messages to actual follow up at the district, and in many cases, the school level. This monitoring allowed these programs to communicate the importance for the program to teachers and to help the government program implementers to respond to program challenges and improve implementation fidelity and quality. One of the effective Learning at Scale program participants noted that “once you monitor, people will implement. If you stop monitoring, people will stop” (Stern et al., 2021).

Fourth, these effective large-scale programs built capacity for the purpose of a gradual transfer of responsibility. This meant that government leaders were actively engaged in essential portions of implementation. This includes a variety of structured pedagogy program subcomponents, including the materials development and teacher professional development sections described below, but also the broader work of coaching, evaluation, and assessment. This transfer of responsibility to government leaders was essential to allow the programs to implement on their own, embedded into the daily instructional program.

Fifth, the institutionalization of these activities was shown to be an essential element of why these programs were successful. Various elements of the programs were more or less effective at long-term institutionalization, but efforts to make the structured pedagogy programs a part of the normal activities of government officers is essential to implementation. More details on how these structured pedagogy programs were implemented, and specifically systems, is found in Stern et al. (2021).

### 5.2 Structured pedagogy instructional materials

The previous section presented evidence about how effective structured pedagogy programs work at scale. In this section we present descriptive ideas as to how materials can be developed, as this is an essential part of what differentiates structured pedagogy programs from the status quo. The evidence presented below is drawn from Dubeck and Sitabkhan (2020) and Mejia and Ralaingita (2020). Designing structured pedagogy instructional materials is an informed, collaborative, and iterative process. Experience in LMICs suggests that the process of a needs assessment through print-ready materials requires a minimum of 6–12 months. Additional time should be allocated to pilot the materials and adjust based on what was learned from the pilot and work with government bodies for formal approvals. Once the materials are print ready, plans need to be made to ensure adequate time for printing before the start of the upcoming school year.

The design process begins with understanding the existing government curriculum. This means studying its strengths and the views of teachers on the curriculum. This evidence can be collected from interviews with the curriculum department, reviewing curriculum documents, teacher interviews, and classroom observations. This analysis should be led by a literacy or numeracy expert.

Working with the government leaders in relevant areas such as curriculum, policy and teacher education, a small team is formed to make key technical decisions. The initial decisions relate to structural options, including which grades, subjects, languages, learning proficiencies, and material types (e.g., a teacher’s guide, student textbook and supplementary readers) are required. Agreement on level of scaffolding to provide teachers is another structural decision, which is informed by the discussion around scripting we described above. Decision-makers need to make initial content choices as well, including guiding principles such as the length of words or texts.

After these initial choices, the small technical team develops a scope and sequence that plots out the skills and content that the students learn each day and week. The scope and sequence adapts the curriculum into a document that maps out the entire structured pedagogical program systematically. The scope and sequence document serves as a map for writing the textbooks and the teacher guides and will be an iterative document, continually updated during the materials-writing process.

A successful structured pedagogy materials production team has clear roles assigned each member. This team includes pedagogical expert for numeracy or literacy language experts, reviewers, writers (including teachers), graphic designers, a production manager, and dedicated administrative support. Although filling each of these roles is critical, engaging too many people under the assumption it will increase the production speed risks lowering the quality of the materials.

The sequential order of developing the student book and the teacher’s guide is not fixed. Developing them simultaneously has advantages but requires substantial coordination skills to avoid errors. So, ideally, the production schedule of the student book and teacher guide is somewhat staggered. Relatedly, design decisions must begin early to establish parameters of the page layout and to avoid writing either too little or too much content per lesson. In addition, choices need to be made about the instructional routines that should be used regularly and an activity bank established with clear direction on how many times each instructional routine is used in a week. During the materials-writing process, the scope and sequence serves as the guiding map combined with other materials providing support to content development and adaptation. The literacy or numeracy expert will give technical explanations intermittently to ensure that all writers understand the purpose of skills being developed in the scope and sequence. Writers will create a full unit of lessons and share the structure with the graphic designer with the goal to have everything clearly laid out for the teacher in a repeatable, recognizable manner. The teams who are providing the reviews work so that revision can be ongoing.

Since the goal of structured pedagogy instructional methods is to support students so they can effectively apply the skills independently, the teacher’s guides will provide structure to the skills required to support this evolution. The gradual release model is one option to provide this scaffold, where discrete skills are taught with a consistent structure of modeling, guided practice, followed by independent practice. That structure is useful for skills such as learning letter sounds or other discrete skills. For higher order skills, the instructional activities will use a gradual release model, but it won't be linear. For example, when learning to solve math problems, students should explain the solutions they used while the teacher summarizes and condenses the explanations for other classmates to use. Subsequently, students will be assigned to apply the solution structures to similar problems on their own while the teacher monitors. Oscillating between student problem attempts and teacher explanation, even though nonlinear, involves the teacher guiding the student with enough scaffolding to eventually release them to do the skill on their own.

### 5.3 Structured pedagogy program training and ongoing support

Complementing the coordinated teacher guide and student books will be a coordinated teacher-support system. This will help teachers learn to effectively use the materials initially, to improve their use over time, and to build skills automaticity. The methods described here are derived in part from the structured pedagogy resources on teacher training and teacher support (Mejia, 2020, Ralaingita, 2020). Evidence from Indonesia shows that suggests that the typical teacher training and support system’s inability to improve learning outcomes is due to a failure to consider that the end goal of this teacher support system is teacher pedagogical improvement rather than simply low-cost coverage (Revina et al., 2020). Given the complexity of helping teachers change their instructional methods, teachers need teacher training and support programs designed to help them through that process, and many typical interventions are simply not designed that way.

#### 5.3.1 Teacher training

After quality materials are developed, the structured pedagogy program focuses on providing high-quality in-service teacher training. Based on the design of the program and the learning materials, the training content should consider what skills teachers in this context already have and prioritize what they need to learn to feel comfortable in trying the unfamiliar parts of the structured pedagogy program. The training will be more effective if it is designed to support the new pedagogical skills that teachers need to do immediately upon returning to their classroom at the end of the teacher training.

To ensure consistency across training sites, a training manual should be developed that is concise, well organized visually, and direct in its reference to the teachers’ guide and other instructional materials. Using examples from the actual teacher guide will demonstrate the expectation that teachers should use the teacher guides daily. The manual needs a detailed but realistic agenda that allocates time for each activity along with buffer time so that all the training content can be covered.

Selecting trainers is an essential decision in a long list of logistics needed to organize a training implemented at large scale (Mejia, 2020). Trainers should include government officers who have the skills to provide the pedagogical methods. All trainers should attend a focused training of trainers where they experience the training they will eventually deliver. Based on the number of teachers to be supported, it might make sense to use a cascade model, where there are subsequent levels of training. Ideally, or if possible, one should maintain a ratio of two trainers to thirty teachers so that feedback and interaction is plentiful (Mejia, 2020). One person who attends the training serves as quality assurance, ready to make adjustments and clarifications when necessary. Teachers should not be expected to train colleagues who did not attend back at their home schools. Head teachers or principals should be provided an abbreviated version of the training and provided with the teachers’ guide so they understand the expectations for their teachers and can reinforce the program at the school level. Decisions whether to implement a residential or nonresidential training are informed by multiple factors, including costs. In many contexts, residential trainings are more expensive and not necessarily more effective if clustered sets of schools can be used for nonresidential training.

Research on large-scale teacher training suggests that teacher training methods will include a mix of teacher practice, skills modeling, discussion, and minimal lecture (Piper et al., 2021). Teachers practicing in dyads and triads should dominate the practice time. Large group or whole group methods can be used to model pedagogical activities for teachers. The modeling must be high quality and ideally done by a dynamic, skilled teacher. Discussion time is useful to clarify the methods and provide for self-reflection or self-evaluation. Lecture should only be minimally used so that teachers maximize the available time to improve their skills.

#### 5.3.2 Ongoing support

Even successful initial teacher trainings require targeted, ongoing support to ensure that teachers understand how to use the materials and that fidelity of implementation is high. The ongoing support that teachers receive from a coach, head teacher, or education officer can influence their motivation to implement the intervention consistently and, therefore, the likelihood they will correctly implement the structured pedagogical lessons and see student learning increase.

Selecting the model of ongoing teacher support is a key step in implementing structured pedagogical programs. Multiple teacher-support models exist in LMICs, and a combination of modalities can be an effective option, costs allowing. In Table 2, we present the pros, cons, and best practices of each model. Regardless of the specific model selected, all teachers implementing a structured pedagogical program need to be observed, given constructive feedback, and get a chance to reflect on their experiences.

**Table 2:** Teacher support modalities (Ralaingita, 2020).

**Modality**	**Pros**	**Cons**	**Best Practice**
**In-school coach**	Inexpensive. Allows for frequent observation and feedback. Can help ensure school-level commitment.	School administrators may be too overloaded to handle this role. Difficult to monitor. May involve extra training and support for school staff.	• Mentorship, not inspection, is focus • Simple observational tools to address instruction • Phased expectations^a^ • Identify clear, specific, and actionable goals • Brief student assessments
**External coaching visits**	Coaches can offer high-level training and can be a conduit for other experts to provide additional information.	Expensive. If coach-to-school ratio is high, or if travel is difficult between schools, teachers may receive few visits.	
**School-level teacher learning groups**	Inexpensive. Can create a positive school environment for trying new approaches.	Less effective if only a few teachers per school. Without enough support, meetings can lose focus or reinforce misconceptions.	• Training and support for facilitation • Pedagogical concerns prominent • Example activities and agenda suggested • Balance structure and individual concerns • Technical experts join occasionally
**Cluster-level teacher learning groups**	Relatively inexpensive and can energize teachers. Can be effective for finding solutions to problems or issues.	Need time and a budget for teachers to meet. Also need support and technical input to ensure that joint solutioning is technically sound.	
**Support via digital technology**	Help to bridge gaps where frequent in-person communication is not possible, or where an expert cannot visit all schools frequently.	Most effective combined with other approaches. Connectivity and access to digital devices must be considered.	• Coaches send reminders and tips via text messages • Community of practice via virtual platforms (e.g., Viber, WhatsApp) • Learning modules and interactive dashboards using interactive voice response and online learning platforms

a Begin with instructional behaviors that teachers can master before adding additional ones for them to use.

As a teacher-support system is being selected, consider the goal of sustaining the support structure utilizing government resources only. By using operational research and monitoring, the system will be able to identify what is working and what needs adjusting. The budget outlays applied in the teacher-support system should be realistic, but not so low that it is ineffective at providing the support teachers need. Where possible, the instructional support mechanisms should be done by existing government-funded personnel. If, as is the case in many countries, the staff member providing pedagogical support has a supervisory or punitive role; more training will be needed to guide them on how to become a supportive technical officer.

## 6 Data systems and accountability in structured pedagogy programs

A structured pedagogy program collects and uses data at several key points. Beginning with data to advocate for the program, followed by data to inform the design of the program, data is essential in structured pedagogy program design choices. But once the program is active, the importance and prevalence of ongoing data collection and a means to access it increases, as it serves to understand the program's influence and ways the program can be improved. The goal of the structured pedagogy data system is to understand how teaching and learning is changing and how that change is affecting children's literacy and numeracy outcomes (Stern and Jordan, 2020).

### 6.1 Choosing which data to collect

Determining the data to be collected is relatively straightforward. After developing a theory of change for the structured pedagogy program, the focus of the program data collection is to measure the key portions of the theory of change, including the key inputs and essential outputs. It is essential to develop a learning agenda of research questions to understand whether the program is being implemented as intended. This could include in-depth case studies examining coaches implementing with fidelity or conducting small A/B testing quantitative comparisons within the larger program to determine which program choice is most effective including teachers’ reactions to the instructional materials (e.g., is it an appropriate amount of detail in the lesson plan?).

### 6.2 How and when to collect the data

From the onset, embedding data collection within existing government systems is an important step as it reduces data duplication or the tendency to develop parallel systems and it increases government leadership. In collaboration with government, and guided by the theory of change, conduct a backward mapping of data needed and what is already being collected. When doing this, map the frequency data will be collected and when it will be needed to inform adaptation and action. Identify technologies, such as tablet-based data collection and a daily updated data dashboard, that will facilitate timely data collection, analysis, and use through government structures. If there is concern that elements such as training, installation, and updating the technology cannot be maintained by government, prioritize the technology that allows for information to be shared between locations to understand what is happening across the program. Recently, the Tusome program transitioned the tablet-based monitoring system to the national government, allowing for the data collection system to move beyond the lower primary levels and literacy areas only.

Determining who collects the data includes understanding who is responsible for delivery of each element of the program. Specifically, examine the lines of external, internal, and organizational accountability to help ensure that the data are not biased by who is collecting it. For example, teachers and head teachers at the school level should not be used for impact evaluations where independence and reliability are critical for data validity.

### 6.3 Using and accessing the data

Data are only valuable if they are used to effect change and improve program quality. To help create a feedback loop, several actions are required. Existing instruments should be updated to ensure they measure quality of structured pedagogy program implementation. There needs to be reliable and ongoing paths for data analysis and reporting at different levels of the decentralized system. In other words, not everyone needs full access to the entire data set; government officers need access to the data in areas that will be influential for decision-making at their level. Available data presented in simplified ways can increase accountability for the behavior of civil servants. Structured pedagogy programs can facilitate regular events at the national and subnational level to share data and results. Using data to improve program implementation and effectiveness of a particular officer’s job will build demand for using data to improve education performance as a regular feature of the system.

This section presented the key characteristics of structured pedagogy programs, including how they work with government, how their materials are developed, how teachers are trained and supported, and how structured pedagogical program’s monitoring and accountability systems can work.

## 7 Limitations and additional research required

The evidence on structured pedagogy programs is growing, but there remain several areas of limited evidence or where additional research is needed. In this section we discuss the resistance to structured pedagogy programs, the relationship between learning outcomes and GDP growth, the duration of structured pedagogy program support, and the need for increased cost-effectiveness research on the impacts of structured pedagogy programs.

Structured pedagogical programs have critics, including in LMICs. For example, Bridge International Academies have been criticized by a teachers’ union for its scripted tablet-based lessons, though they have reduced the scripting of those lessons over time (Riep, 2019). Some educators are concerned about how some structured pedagogy programs are perceived to be designed to be teacher-proof and that the existence of the structured materials can be seen as deprofessionalizing their work (Datnow and Castellano, 2000). The resistance toward structured pedagogy programs may emanate from the leadership of teachers’ unions, curriculum bodies, or other parts of government. This resistance is related to whether structured pedagogical programs work, but also that these programs have been developed in the West and pushed toward LMICs without a careful understanding of these contexts or the experiences of teachers in those locations.

The evidence we have presented suggests that structured pedagogy programs can have an impact on learning outcomes, but there are still some outstanding questions about their use.

It is worth examining whether the structured pedagogy programs provision of teachers’ guides with lesson plans linked to student books should be provided as a short-term scaffold for struggling education systems, or whether a long-term support system to improve learning is the better approach. Mourshed et al. (2010) argued that structured pedagogy programs should be focused on the process of helping systems that are characterized as *poor* to increase to *fair*. Their claim is that having tight control of teaching and learning is essential to dramatically improve learning in these struggling contexts but that other solutions might be better suited for systems that are better performing. Given the meaningful impacts of structured pedagogy programs in many LMICs, it is not clear how the structured pedagogy programs that are increasing outcomes should continue after a system has reached a certain level. As we show in Fig. 2, teacher decision-making occurs along a continuum, and it may be that the structured pedagogy program can be adjusted to provide more autonomy within the implementation of a program as suggested by Global Education Evidence Advisory Panel (GEEAP) (2023). It seems important to explore this, as several programs seem to have program effects plateau after initial gains. Countries that are moving toward skills- or competency-based curricula may have a different definition of teacher autonomy and are somewhat more resistant to the structured approaches discussed here. On the other hand, beginning teachers tasked with teaching a subject where they have limited pedagogical content knowledge would continue to benefit from structured pedagogy programs. It may be that improvements in the quality of pre-service teacher training would reduce the need for teacher training and ongoing support and only need the instructional materials. In short, the duration of support from structured pedagogical interventions is an empirical unknown and should be answered by research evidence.

While structured pedagogy programs have increased in LMICs, the evaluations and studies of those programs have not typically included cost-effectiveness estimates. The Smart Buys report characterized the handful of structured pedagogy programs by their cost-effectiveness and found that some of these programs were as cost-effective as any other program in the education sector, averaging more than three learning-adjusted years of schooling per $100 investment (Global Education Evidence Advisory Panel (GEEAP), 2023). More effort is required to learn more about how costs are allocated and whether different cost allocations result in greater cost-effectiveness. This is critical data to share with policymakers as they consider how to allocate their resources.

## 8 Conclusions

Structured pedagogy programs are expanding in LMICs as a method to respond to the learning crisis alongside of TaRL programs. Particularly in the context of substantial learning loss due to COVID-19, more work is required to identify locally implemented solutions to improve learning outcomes, including structured pedagogy (Angrist et al., 2021). Given the potential effectiveness of these programs, more evidence is required to understand whether and under what conditions these programs work in various contexts.

## CRediT authorship contribution statement

**Benjamin Piper:** Conceptualization, Formal analysis, Writing – original draft. **Margaret Dubeck:** Conceptualization, Formal analysis, Writing – original draft.

## Conflicts of interest statement

We wish to confirm that there are no known conflicts of interest associated with this publication and there has been no significant financial support for this work that could have influenced its outcome.
